# Co‐evolutionary dynamics between a defensive microbe and a pathogen driven by fluctuating selection

**DOI:** 10.1111/mec.13906

**Published:** 2016-12-05

**Authors:** Suzanne A. Ford, David Williams, Steve Paterson, Kayla C. King

**Affiliations:** ^1^ Department of Zoology University of Oxford The Tinbergen Building South Parks Road Oxford OX1 3PS UK; ^2^ Centre for Genomic Research Institute of Integrative Biology University of Liverpool Biosciences Building Liverpool L69 7ZB UK

**Keywords:** co‐evolution, defensive mutualism, experimental evolution, fluctuating selection dynamics, pathogen

## Abstract

Microbes that protect their hosts from pathogenic infection are widespread components of the microbiota of both plants and animals. It has been found that interactions between ‘defensive’ microbes and pathogens can be genotype‐specific and even underlie the variation in host resistance to pathogenic infection. These observations suggest a dynamic co‐evolutionary association between pathogens and defensive microbes, but direct evidence of co‐evolution is lacking. We tested the hypothesis that defensive microbes and pathogens could co‐evolve within host populations by co‐passaging a microbe with host‐defensive properties (*Enterococcus faecalis*) and a pathogen (*Staphylococcus aureus)* within *Caenorhabditis elegans* nematodes. Using both phenotypic and genomic analyses across evolutionary time, we found patterns of pathogen local adaptation and defensive microbe–pathogen co‐evolution via fluctuating selection dynamics. Moreover, co‐evolution with defensive microbes resulted in more rapid and divergent pathogen evolution compared to pathogens evolved independently in host populations. Taken together, our results indicate the potential for defensive microbes and pathogens to co‐evolve, driving interaction specificity and pathogen evolutionary divergence in the absence of host evolution.

## Introduction

Many microbes can determine host health by defending against pathogenic infection (Ford & King 2016). Such ‘defensive’ microbes are a valuable addition to the host immune system (Gerardo & Parker 2014) and are found in a diversity of plants and animals, including humans (Ford & King 2016). It is theoretically predicted that defensive microbes and pathogens could undergo co‐evolutionary interactions within a host via fluctuating selection dynamics (Kwiatkowski *et al*. 2012). Consistent with this prediction, interactions between defensive microbes and pathogens in nature (including parasites and parasitoids) can show a high degree of specificity and determine the variation in host resistance to pathogenic infection (Oliver *et al*. 2005; Poulsen *et al*. 2010; Koch & Schmid‐Hempel 2012; Rouchet & Vorburger 2012; Cariveau *et al*. 2014; Cayetano & Vorburger 2015). These observations suggest the potential for a dynamic co‐evolutionary association between defensive microbes and pathogens within host populations. However, direct tests of defensive microbe–pathogen co‐evolution are currently lacking (Ford & King 2016).

Co‐evolutionary pattern and process can vary across interacting species (Gaba & Ebert 2009; Brockhurst & Koskella 2013), with co‐evolution occurring via arms race dynamics or fluctuating selection dynamics. Arms race dynamics can occur when interacting species accumulate generally beneficial traits that can become increasingly exaggerated (Agrawal & Lively 2002). Alternatively, fluctuating selection dynamics occur when traits are advantageous only when rare in the population, resulting in cycles of genotypes through time (Agrawal & Lively 2002). Importantly, this rare advantage only results when there is a high degree of specificity in species interactions. As such, the observed specificity in defensive microbe–pathogen interactions in nature (Ford & King 2016) indicates the possibility of co‐evolution via fluctuating selection dynamics, consistent with theory (Kwiatkowski *et al*. 2012).

**Figure 1:**
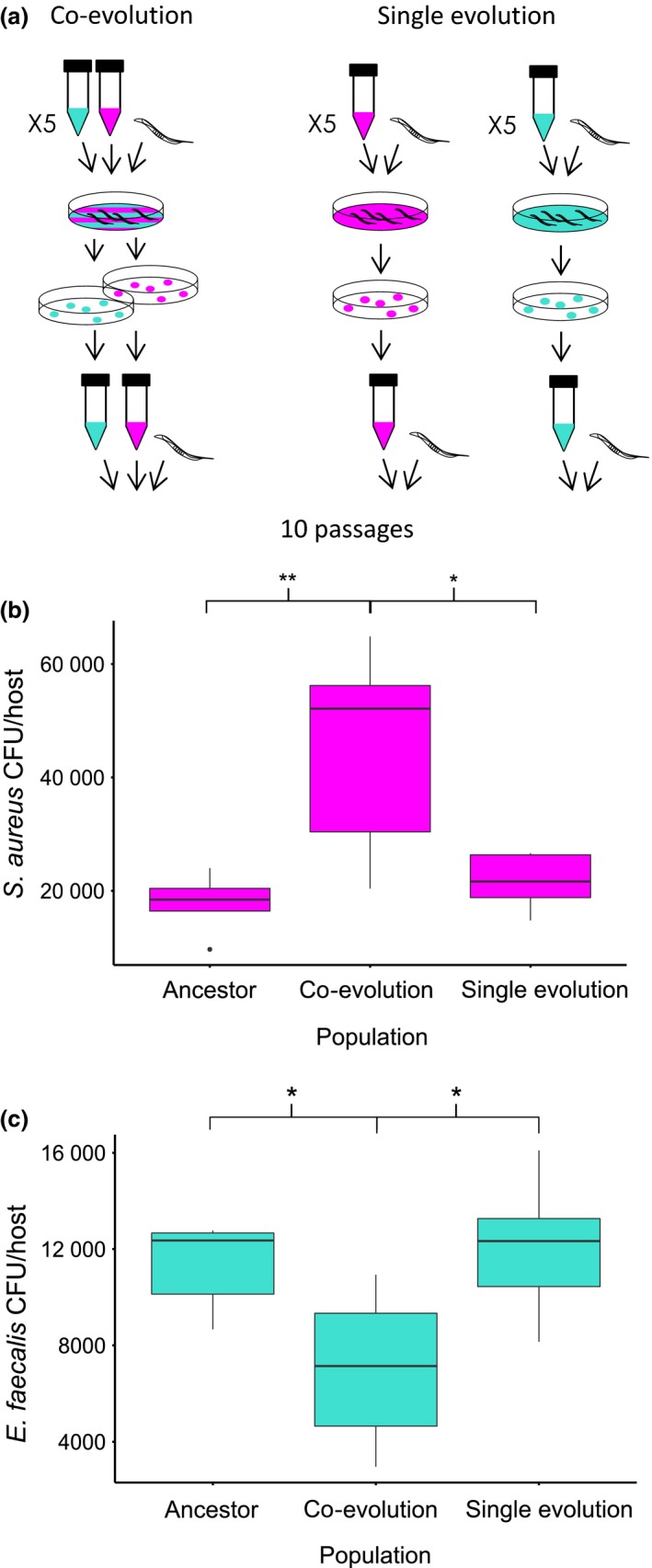
Experimental co‐evolution. (a) Design of evolution experiment: *Staphylococcus aureus (magenta)* and *Enterococcus faecalis* (turquoise) were co‐passaged (co‐evolution) or passaged independently (single evolution). At each passage, the same genetically homogeneous population of *Caenorhabditis elegans* hosts was exposed to *S. aureus* and/or *E. faecalis*. After 24 h of exposure, 10 colonies of *S. aureus* and/or *E. faecalis* were collected from the pooled guts of 10 hosts using selective media and passaged to the next generation. Each treatment had five replicate populations and was conducted for 10 passages. (b) Fitness of *S. aureus* (CFU/host) under co‐colonization with *E. faecalis*. Populations of *E. faecalis* and *S. aureus* were co‐colonized from the same replicate populations in each treatment. (c) Fitness of *E. faecalis* (CFU/host) under co‐colonization with *S. aureus*. Populations of *E. faecalis* and *S. aureus* were co‐colonized from the same replicate populations in each treatment.

In this study, we experimentally co‐evolved a microbe with host‐protective properties (*Enterococcus faecalis*) and a pathogen (*Staphylococcus aureus*) within nonevolving *Caenorhabditis elegans* host populations (co‐evolution treatment, Fig. 1a). We also passaged the defensive microbe and pathogen independently within *C. elegans* populations (single evolution treatment, Fig. 1a). Each treatment consisted of five replicate populations originating from the same clone of each bacterial species such that any evolutionary change was de novo. We conducted the evolution experiment for 10 passages, by which time considerable evolutionary change is known to occur in this system (King *et al*. 2016). *Caenorhabditis elegans* is used as a model for investigating microbial pathogenesis and host–microbiota interactions (Gravato‐Nobre & Hodgkin 2005; Cabreiro & Gems 2013; Hodgkin *et al*. 2013; Clark & Hodgkin 2014; Gray & Cutter 2014). Both *E. faecalis* and *S. aureus* are found in the animal microbiota and can colonize the gut of *C. elegans* (Garsin *et al*. 2001). In this experiment, we used bacteria isolated from humans. As a result, their interaction within a nematode host is novel such that we can explore the evolutionary dynamics of defensive symbioses early in their formation. We have previously shown that within‐nematode interactions between *E. faecalis* and *S. aureus* mimic those of a defensive microbe and pathogen, respectively (King *et al*. 2016). *Enterococcus faecalis* can suppress *S. aureus* fitness and virulence by producing antimicrobial superoxides (King *et al*. 2016). At the same time, *E. faecalis* also exploits *S. aureus* by thieving the iron‐scavenging siderophores it produces (Ford *et al*. 2016). Importantly, both superoxide production by *E. faecalis* and siderophore production by *S. aureus* have been shown to evolve under co‐colonization, indicating the possibility of co‐evolutionary interactions (King *et al*. 2016; Ford *et al*. 2016).

By experimentally co‐passaging *E. faecalis* and *S. aureus* within *C. elegans* hosts, we found direct evidence of defensive microbe–pathogen co‐evolution. We revealed that the defensive microbe and the pathogen underwent reciprocal adaptation via fluctuating selection dynamics and produced patterns of pathogen local adaptation and genetic diversification. Our results support theoretical predictions and illustrate the potential for defensive microbes to drive patterns of pathogen adaptation in the absence of host evolution.

## Materials and methods

### Nematode host and bacteria

*Caenorhabditis elegans* nematodes constantly interact with microbes in their natural habitats (Félix & Braendle 2010), wherein they can act as predators or hosts for numerous microbial species (Clark & Hodgkin 2014; Petersen *et al*. 2015). These animals are thus an established model for microbial colonization and pathogenesis (Gravato‐Nobre & Hodgkin 2005; Cabreiro & Gems 2013), and their gut can be co‐colonized by multiple pathogens and commensals (Berg *et al*. 2016; Dirksen *et al*. 2016; Samuel *et al*. 2016).

We used the simultaneous hermaphroditic N2 wild‐type *C. elegans* strain from the *Caenorhabditis* Genetics Centre (University of Minnesota, Minneapolis, MN). A genetically homogeneous line was generated by selfing a single hermaphrodite for five generations. Populations of these nematodes were frozen in 50% M9 solution and 50% liquid‐freezing solution in cryotubes at −80 °C (Hope 1999). Populations were regularly resurrected to prevent the accumulation of de novo mutations in hosts. Nematodes were maintained at 20 °C on nematode growth medium (NGM) plates seeded with *Escherichia coli* OP50. Seeded NGM was made by growing *E. coli* OP50 at 30 °C shaking at 200 rpm overnight in Luria‐Bertani (LB) broth and then incubating 100 μl of this culture on NGM plates at 30 °C overnight (Hope 1999). To ensure clean stocks for experimentation and to synchronize the age of individuals in the population, nematodes were regularly treated with a bleach (NaClO) and sodium hydroxide (NaOH) solution that kills both microbes and worms but not worm eggs (Hope 1999).

We used *Staphylococcus aureus* strain MSSA 476 (GenBank: BX571857.1), an invasive community‐acquired methicillin‐susceptible isolate, and *Enterococcus faecalis* strain OG1RF (GenBank: CP002621.1), an isolate from a human digestive tract. A single ancestral population of each species was grown from a single colony overnight in Todd‐Hewitt Broth (THB) shaking at 200 rpm at 30 °C. Bacteria were frozen in a 1:1 ratio of sample to 50% glycerol solution in cryotubes at −80 °C.

### Experimental co‐evolution

The experiment consisted of two treatments: (i) *S. aureus* and *E. faecalis* were co‐passaged under co‐colonization within host populations (co‐evolution) and (ii) *S. aureus* and *E. faecalis* were passaged separately in host populations (single evolution) (Fig. 1a). Each treatment consisted of five replicate populations and 10 passages. To make exposure lawns, bacteria were cultured overnight in THB shaking at 200 rpm at 30 °C. After standardizing the cultures to an OD600 reading of 1.00, the bacteria were spread onto Tryptic Soy Broth (TSB) plates. We spread a volume of 120 μl of either *S. aureus* or *E. faecalis* for the single evolution treatment, and we spread a mixture of 120 μl of *S. aureus* and 120 μl of *E. faecalis* on the same exposure plate for the co‐evolution treatment. Exposure plates were kept overnight at 30 °C.

Approximately 1000 young adult nematodes from the stock *C. elegans* population were placed onto each exposure lawn and incubated at 25 °C. After 24 h of incubation, bacteria from 10 dead nematodes from each lawn were picked to ensure that we took hosts successfully colonized with bacteria. Under co‐colonization, the proportion of pathogen collected from the infection did not differ significantly between dead and live worms (Ford *et al*. 2016). Nematodes were considered dead when they did not respond to touch with a platinum wire. Nematodes were washed to remove external bacteria by transferring them between five 5 μl drops of M9 buffer on fresh TSB plates using a platinum wire. Nematodes were then crushed in 20 μl of M9 in a 1 ml Eppendorf tube with a pestle to release internal bacteria. An inoculation loop was used to streak each sample onto selective media (TSB plates with 100 μg/ml rifampicin were used to isolate *E. faecalis,* and Mannitol Salt Agar (MSA) plates were used to isolate *S. aureus*), and the plates were incubated overnight at 30 °C. Ten colonies of *S. aureus* and/or *E. faecalis* were subsequently picked from each replicate and grown in THB overnight, shaking at 200 rpm at 30 °C. These cultures were used to make the exposure plates for the next passage.

### Co‐colonization dynamics

To determine whether co‐passage affected the fitness of the defensive microbe and pathogen under co‐colonization, we compared the fitness of the defensive microbe and pathogen from the Co‐evolution treatment under co‐colonization with the ancestral and single evolution treatment populations under co‐colonization. We performed this experiment using populations from passage 10 of the evolution experiment. Exposure plates were made as above. Approximately 1000 young adult nematodes from the stock *C. elegans* population were placed onto exposure plates and incubated for 24 h at 25 °C. After 24 h of exposure, three to five dead nematodes were picked from each plate. Nematodes were washed as in the evolution experiment and then crushed in 20 μl of M9 with a pestle to release internal bacteria. After crushing, serial dilutions were plated onto selective media and grown overnight at 30 °C. *E. faecalis* and *S. aureus* colony‐forming units (CFUs) per host were counted.

### Local adaptation in time

**Figure 2:**
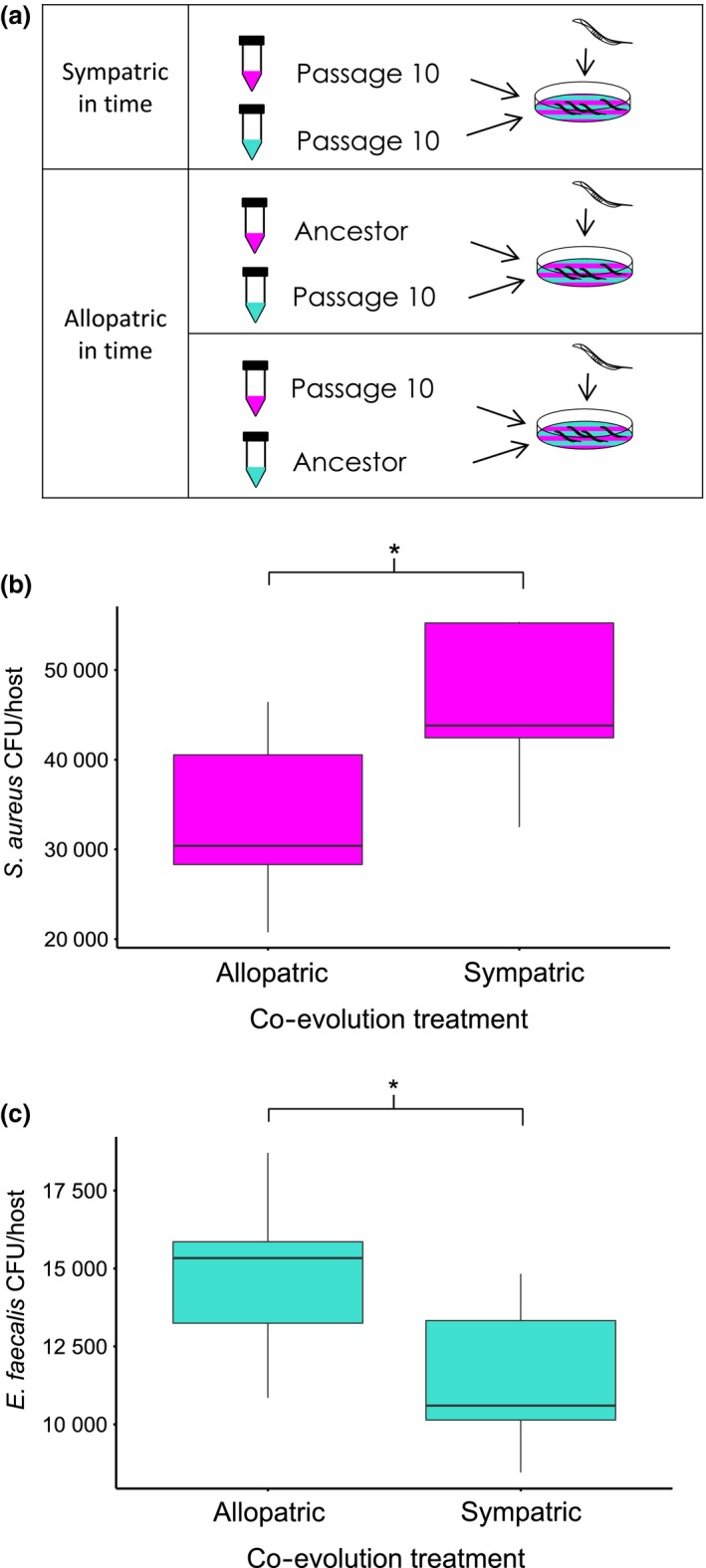
Local adaptation in time. (a) Sympatric‐ and allopatric‐in‐time conditions. Sympatric conditions involve co‐colonization of contemporary *Staphylococcus aureus* (magenta) and *Enterococcus faecalis* (turquoise) from the same replicate population at passage 10. Allopatric crosses involve co‐colonization of ancestral and passage 10 populations. (b) Fitness of *S. aureus* (CFU/host) under allopatric and sympatric conditions (co‐evolution treatment). (c) Fitness of *E. faecalis* (CFU/host) under allopatric and sympatric conditions (co‐evolution treatment).

To test whether the defensive microbe or pathogen became locally adapted in time as a result of co‐passage, we measured the number of CFUs per nematode of each bacterial species under sympatric‐in‐time and two allopatric‐in‐time co‐colonization conditions (see Fig. 2a). Exposure plates were made as above. The sympatric condition paired defensive microbe and pathogen populations from the same replicates at passage 10. The two allopatric conditions paired defensive microbe and pathogen ancestors with passage 10 populations. Our assays of local adaptation in time are conducted in a similar fashion to the ‘local vs. foreign’ and ‘home vs. away’ comparisons commonly used to measure local adaptation in space (Blanquart *et al*. 2013). Using two allopatric conditions in a single comparison is a conservative method of measuring local adaptation because it allows us to tease apart the evolutionary effects of both interacting species. We also performed the same comparisons using the single evolution treatment populations. Given that both bacterial species evolved independently in the single evolution treatment, the sympatric pairings were ‘pseudosympatric’, and we did not expect to find a signal of local adaptation.

### Time shifts

**Figure 3:**
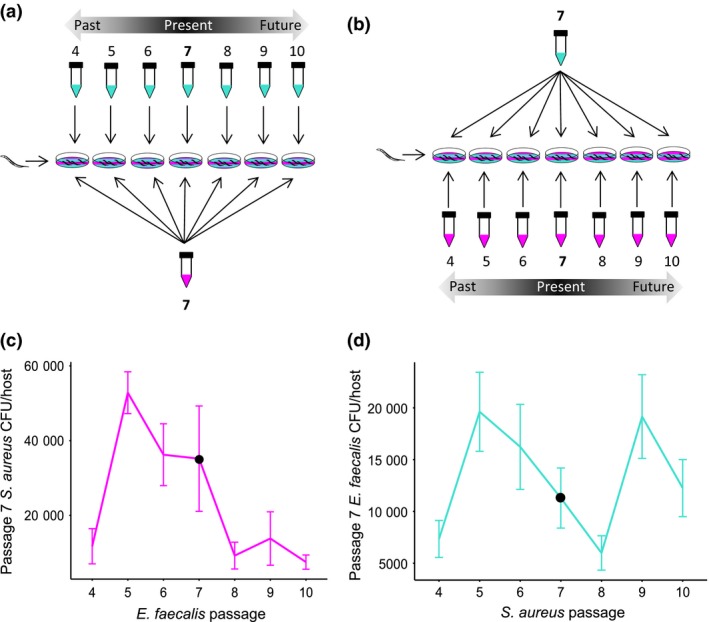
Time shifts. Design of time‐shift assays whereby (a) *Staphylococcus aureus* pathogen (magenta) and (b) *Enterococcus faecalis* defensive microbe (turquoise) replicate populations were paired with the other from the past, present and future time points. (c) Fitness of *S. aureus* (CFU/host) from passage 7 against *E. faecalis* from passages 4–10 (co‐evolution treatment). (d) Fitness of *E. faecalis* (CFU/host) from passage 7 against *S. aureus* from passages 4–10 (co‐evolution treatment).

We looked for evidence of reciprocal adaptation in both pathogen and defensive microbe populations over time by performing time‐shift assays (Gaba & Ebert 2009). Here, *S. aureus* populations from passage 7 were allowed to co‐colonize the host with *E. faecalis* from past, present and future replicate populations (passages 4–10, see Fig. 3a), and their respective CFUs/host were counted. The reciprocal was carried out for *E. faecalis* (see Fig. 3b). Exposure plates were made, and CFU per host for each species was counted as above.

### Genome extraction and analysis

Forty clones were randomly selected from each *S. aureus* and *E. faecalis* replicate population from both selection treatments at passages 5 and 10. Each clone was grown independently overnight in 200 μl THB in a 96‐well plate shaking at 175 rpm at 30 °C. Subsequently, each set of 40 clones was checked for equal OD600 and pooled in equal volumes, resulting in one sample per 40 clones. A single clone of ancestral *S. aureus* and *E. faecalis* was also grown.

*Staphylococcus aureus* DNA was extracted by centrifuging 1 ml of culture at 6000 ***g***, removing the supernatant and resuspending the pellet in 160 μl enzymatic lysis buffer (Qiagen), 40 μl lysostaphin (200 μg/ml, Sigma‐Aldrich), 40 μl lysozyme (100 mg/ml, Sigma‐Aldrich) and 8 μl RNase A (10 mg/ml, Sigma‐Aldrich). This mixture was incubated for 1 h at 37 °C. A volume of 25 μl proteinase K (Qiagen) was then added, and 200 μl Buffer AL (without ethanol) was added and vortexed. This mixture was then incubated at 56 °C for 1 h. A volume of 200 μl of ethanol was added and vortexed and DNA purification followed DNeasy Blood and Tissue Spin‐Column Protocol (Qiagen). From *E. faecalis*, DNA was extracted by centrifuging 2 ml of culture at 7500 rpm, removing the supernatant and resuspending the pellet in 160 μl buffer B1 (Qiagen), 6 μl of 25KU/ml mutanolysin (Sigma‐Aldrich), 40 μl of 200 μg/ml lysostaphin (Sigma‐Aldrich) and 40 μln 100 mg/ml lysozyme (Sigma‐Aldrich). This mixture was incubated for 1 h at 37 °C. A volume of 25 μl proteinase K (Qiagen) was then added, and 200 μl Buffer AL (without ethanol) was added and vortexed. This mixture was then incubated at 56 °C for an hour. Ethanol (200 μl) was added and DNA purification followed the DNeasy Blood and Tissue Spin‐Column Protocol (Qiagen).

DNA was sequenced using the HiSeq4000 platform with 100‐bp paired end at the Wellcome Trust Centre for Human Genetics. The project accession no. at the European Nucleotide Archive for the raw read data is PRJEB13385. Sequenced read data in fastq files were trimmed for the presence of Illumina adapter sequences using Cutadapt version v1.9.1 (revision e960cc1 from github.com/marcelm/cutadapt) (Martin 2011). The reads were further trimmed using Sickle version 1.33 (revision f3d6ae3 from github.com/najoshi/sickle) (Joshi & Fass 2011) with a minimum window quality score of 25 and retaining only reads longer than 50 bp after trimming. Single reads remaining from pairs were retained.

Reference genomes for short‐read mapping were obtained from the NCBI Assembly database (http://www.ncbi.nlm.nih.gov/assembly). Short reads of the DNA extractions from the *Staphylococcus aureus* strain MSSA476 isolates were mapped to assembly GCF_000011525.1 using BWA MEM v0.7.12 (revision cc9eef2 from github.com/lh3/bwa) (Li & Durbin 2009) using option ‐M to flag shorter split hits as secondary ensuring the single best alignment was used. Evolved populations of *E. faecalis* OG1RF were mapped to assembly GCF_000172575.2 using the same aligner and options. Alignments were manipulated using SAMTools v1.2 (revision ac5b8e7 from github.com/samtools/samtools) (Li *et al*. 2009).

The Genome Analysis Toolkit v3.4 (GATK) (McKenna *et al*. 2010; DePristo *et al*. 2011; Van der Auwera *et al*. 2013) IndelRealigner module was used to realign reads around putative insertions and deletions after which duplicate reads were identified and removed with Picard v1.135 (Carey 2015). Single‐nucleotide polymorphism, insertion and deletion discovery was performed with GATK's Haplotype caller module with sample ploidy *n* = 40 as described in Williams *et al*. (2015). Default parameter values were used, however, given diversity was expected to be relatively low due to the experimental duration the priors ‘–heterozygosity 0.0001 –indel_heterozygosity 0.00001’ were used. One replicate population of *E. faecalis* from the co‐evolution treatment at passage 10 (C3.10) had high read coverage depth over one region that caused excessive memory usage. This coverage difference was remedied by setting ‘–downsample_to_fraction 0.8’ to decrease the reads used and ‘–minPruning 2’ to simplify the de Bruijn k‐mer graph used in variant calling. To distinguish low frequency mutations from sites prone to sequencing error, the ancestral clone was sequenced and variants called with ploidy of 40, as above, and any sites exhibiting apparent polymorphism were filtered out of further analyses. Any sites appearing as polymorphic in more than one population were checked by manual inspection in IGV2.

### Genetic distance

A phylogenetic tree was constructed using Euclidean genetic distances (the square root of pairwise differences), which is suggested as an appropriate metric for molecular variation data (Excoffier *et al*. 1992; Paterson *et al*. 2010). It is important to note that a few observed mutations are likely to have arisen in the overnight culture of the ancestral populations used to initiate all populations of all treatments, resulting in some shared mutations from the start of the evolution experiment. Pairwise genetic distances were taken from the distance matrix for statistical analysis. We compared the genetic distances of replicate populations to the ancestor to assess differences in the rate of evolutionary change between treatments. We compared the genetic distances between replicate populations of the same treatments to contrast the diversity in evolutionary trajectories. Finally, to assess differences in rates of genetic turnover of each treatment, we compared the genetic distances of replicate populations between passages 5 and 10.

### Statistical analysis

Data analysis was carried out in R v 3.2.0 (http://www.r-project.org/). Parametric tests were used for all data, which met the required assumptions. These assumptions were checked using the Shapiro test to detect whether data were normally distributed and *F*‐tests to compare the variances of two samples from normal populations. Nonparametric equivalents were used if the data did not meet those assumptions. Outlying data points were detected and removed using the Dixon test. anovas were used to compare bacterial fitness across treatments under co‐colonization, and Tukey contrasts were used for post hoc comparisons. Plots of each anova were checked by eye for model quality. A mixed‐effect model was used to measure local adaptation, including replicate population as a random effect and the sympatric/allopatric treatment as a fixed effect. Another mixed‐effect model was used to measure reciprocal adaptation across time shifts, including replicate population as a random effect and time shift (passage) as a fixed effect. Tukey contrasts were used for post hoc comparisons. Two‐sample *t*‐tests were used to compare pairwise genetic distances of bacterial populations. The Welch two‐sample *t*‐test was used when the assumption of equal variance was broken. The Wilcoxon rank‐sum test was used when the assumption of normality was broken. *P*‐values generated by comparing pairwise genetic distances were corrected for multiple comparisons for each bacterial species using the FDR method.

## Results

### Co‐colonization dynamics

We found a significant increase in pathogen fitness (Fig. 1b, anova *F* = 7.98, d.f.  = 2, *P* = 0.0063, Table S1, Supporting information) and decrease in defensive microbe fitness (Fig. 1c, anova *F* = 4.86, d.f. = 2, *P* = 0.028, Table S1, Supporting information) over time in the co‐evolution treatment that did not occur in the single evolution treatment. These data suggest that *Staphylococcus aureus* was ‘winning’ at passage 10 of experimental co‐evolution with *Enterococcus faecalis*.

### Local adaptation in time

We found that the high pathogen fitness observed at passage 10 in the co‐evolution treatment under co‐colonization was significantly reduced under allopatric conditions (Fig. 2b mixed‐effect model: χ^2^ = 5.82, d.f. = 1, *P* = 0.016). Conversely, the low defensive microbe fitness recorded at passage 10 in the co‐evolution treatment under co‐colonization significantly increased under allopatric conditions (Fig. 2c mixed‐effect model: χ^2^ = 5.79, d.f. = 1, *P* = 0.016). These results thus indicate that the co‐colonization dynamics observed at passage 10 were a result of interaction specificity due to pathogen local adaptation in time and defensive microbe local maladaptation in time.

As expected, there were no significant differences in *E. faecalis* or *S. aureus* fitness between the allopatric and pseudosympatric pairings from the single evolution treatment (Fig. S1a, Supporting information mixed‐effect model: χ^2^ = 1.8, d.f. = 1, *P*‐0.18. Figure S1b, Supporting information mixed‐effect model: χ^2^ = 0.8, d.f. = 1, *P*‐0.36). Thus, the patterns of local adaptation we observe in the co‐evolution treatment cannot be reproduced by evolving the bacteria independently within the host.

### Time shifts

We discovered strong evidence of reciprocal adaptation between *E. faecalis* and *S. aureus* in replicate populations from the co‐evolution treatment. We found that the fitness of *S. aureus* differed significantly when paired with defensive microbe populations from the past, present and future (Fig. 3c mixed‐effect model: χ^2^ = 29.1, d.f. = 6, *P* = 5.819e‐05, Table S1, Supporting information). Specifically, defensive microbes from the future were significantly better at suppressing pathogens than defensive microbes from the past (8 vs. 5, *P* < 0.001), indicating that the defensive microbe adapted to recently experienced pathogen populations. Critically, as we sampled further back into the past, we found evidence that adaptation was driven by fluctuating selection dynamics. Defensive microbes from the distant past were significantly better at suppressing pathogens than defensive microbes from the recent past (4 vs. 5, *P* < 0.001). From this pattern, we can assume that the defensive microbe genotypes common at passage 8 were also common at passage 4 and were thus fluctuating over time.

From the reciprocal time shift, we found that the within‐host fitness of *E. faecalis* differed significantly when paired with pathogen populations from the past, present and future (Fig. 3d mixed‐effects model: χ^2^ = 17.18, d.f. = 6, *P* = 0.0086, Table S1, Supporting information). Future pathogens were significantly better at suppressing defensive microbes than pathogens from the past (8 vs. 5 *P* = 0.011), suggesting that pathogens were also adapting to recently experienced defensive microbe populations. Consistent with the reciprocal time shift above, we discovered that pathogen adaptation was driven by fluctuating selection dynamics. Here, pathogens sampled further back into the past were significantly better at suppressing defensive microbes than pathogens from the recent past (4 vs. 5 *P* = 0.034). We can assume from this pattern that the pathogen genotypes common at passage 8 were also common in the past at passage 4, consistent with the cycle of defensive microbe genotypes we observed in Fig. 3c. In addition to this apparent cycle, we also discovered that pathogens sampled into the distant future were significantly worse at suppressing defensive microbes than pathogens from the near future (9–8 *P* = 0.017). Interestingly, this fluctuation did not correspond with an equivalent fluctuation in *E. faecalis* genotypes in the reciprocal time shift (Fig. 3c), suggesting that the pathogen was leading a new cycle of adaptation to which the defensive microbes had yet to respond. Together, these phenotypic results provide strong evidence of ongoing, reciprocal adaptation between *S. aureus* and *E. faecalis* by fluctuating selection dynamics.

### Co‐evolutionary genomics

**Figure 4:**
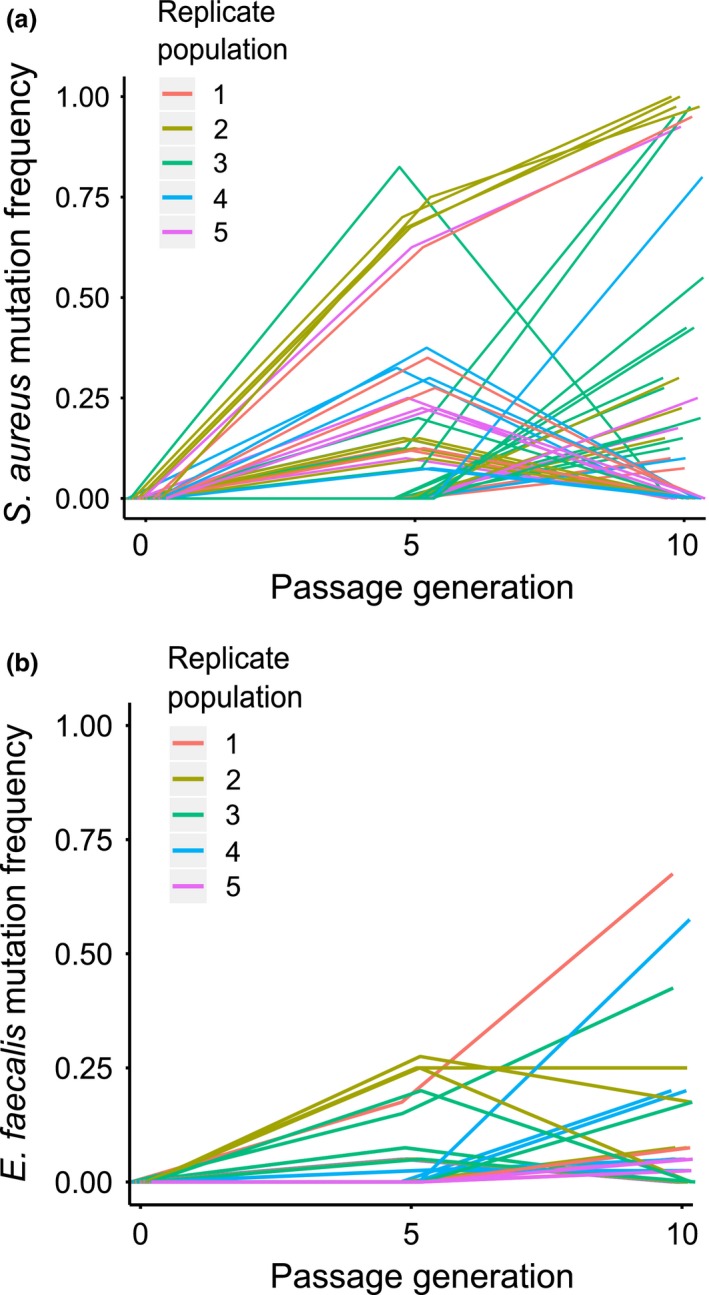
Dynamics of genomic evolution. The frequency of mutations in 40 sampled clones from (a) *Staphylococcus aureus* and (b) *Enterococcus faecalis* replicate populations at passages 5 and 10 under co‐passage (co‐evolution Treatment). Each line represents an individual mutation, and colours correspond to replicate populations.

We plotted the frequency of mutations for each replicate population of the co‐evolution treatment over time for both *S. aureus* and *E. faecalis*. Consistent with the results indicating co‐evolution via fluctuating selection, we discovered that de novo mutations fluctuated in frequency over time in all *S. aureus* (Fig. 4a) and *E. faecalis* (Fig. 4b) replicate populations from the co‐evolution treatment. Most mutations that were common in the populations at passage 5 became rare (or extinct) by passage 10, whilst many rare (or nonexistent) mutations at passage 5 increased in frequency by passage 10. A few mutations were found to increase to near fixation across both time points, indicating either slower fluctuations for these mutations or the existence of simultaneous arms race dynamics.

Several mutations in the co‐evolved *E. faecalis* populations were located in genes with putative functions in the production of superoxides (Huycke *et al*. 2001, 2002), a known protection mechanism in this system (King *et al*. 2016) (See Appendix S1). For example, mutations were found in genes involved in purine metabolism and the transport of xanthine, both of which affect the production of superoxides (Huycke *et al*. 2001, 2002). Interestingly, we discovered the same stop mutation in a gene involved in purine metabolism as evolved in a previous experiment whereby *E. faecalis* had evolved increased superoxide production during repeated exposure to a single *S. aureus* genotype (King *et al*. 2016). In addition to this, many of the mutations in the co‐evolved *S. aureus* populations were located in genes with putative functions in the siderophore pathway, a pathway important in *S. aureus–E. faecalis* interaction (Ford *et al*. 2016) (see Appendix S2). These included putative siderophore‐iron reductases, substrate transporters and proteins involved in glycine metabolism, a key component of ferrichrome (Ford *et al*. 2016).

### Genetic distance

**Figure 5:**
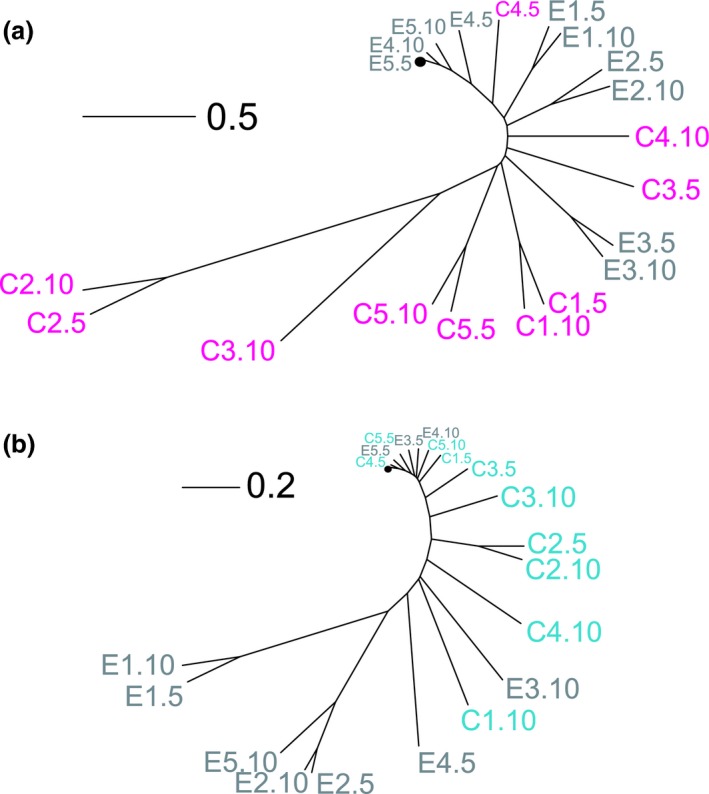
Genetic distances of pathogen and defensive microbe populations. (a) Phylogenetic tree for *Staphylococcus aureus* populations from the single evolution treatment (E1–5 in grey) and co‐evolution treatment (C1‐5 in magenta) from passages 5 and 10 along with the ancestor (black circle). (b) Phylogenetic tree for *Enterococcus faecalis* populations from the single evolution treatment (E1–5 in grey) and co‐evolution treatment (C1‐5 in turquoise) from passages 5 and 10 along with the ancestor (black circle). Genetic distances based on Euclidean distances calculated from the frequency and identity of mutations in each population. Scale bar indicates Euclidean distance.

Co‐passage with the defensive microbe resulted in greater pathogen genetic divergence and turnover than passaging the pathogen through hosts alone. Co‐evolved *S. aureus* populations were significantly more genetically distant from the ancestor and thus experienced faster evolutionary change than independently passaged pathogen populations (Fig. 5a two‐sample *t*‐test, *t* = −2.95, d.f. = 8, fdr‐corrected *P* = 0.018). Furthermore, we found significantly greater pairwise genetic distances between each replicate of the co‐evolved pathogen populations within the independently passaged pathogen populations (Fig. 5a Welch two‐sample *t*‐test, *t* = −6.97, d.f. = 12.9, fdr‐corrected *P* = 3.108e‐05), suggesting that co‐evolving pathogen populations followed more diverse evolutionary trajectories. Co‐evolved *S. aureus* populations also exhibited greater genetic distance between passages 5 and 10 than the independently passaged populations (Fig. 5a Wilcoxon rank‐sum test W = 25, fdr‐corrected *P* = 0.012), indicating greater genetic turnover in the co‐evolved pathogens, consistent with fluctuating selection dynamics.

In contrast, we saw no difference in genetic divergence and turnover between *E. faecalis* treatments. Neither the genetic distance from the ancestor nor the pairwise genetic distances among replicate defensive microbe populations differed significantly between the single evolution and co‐evolution treatments (Fig. 5b genetic distance from ancestor: two‐sample *t*‐test, *t* = 1.1, d.f. = 8, fdr‐corrected *P* = 0.302; among‐replicate genetic distances: two‐sample *t*‐test, *t* = 2.36, d.f. = 18, fdr‐corrected *P* = 0.09). In addition, the co‐evolution treatment did not differ from the single evolution treatment in genetic distance between passages 5 and 10 (Fig. 5b two‐sample *t*‐test *t* = 1.55, d.f. = 8, fdr‐corrected *P* = 0.24).

## Discussion

It is theoretically predicted that defensive microbes and pathogens could undergo co‐evolutionary interactions within host populations via fluctuating selection dynamics (Kwiatkowski *et al*. 2012); however, direct tests are currently lacking (Parker *et al*. 2011; Ford & King 2016; Hahn & Dheilly 2016). Here, by experimentally co‐passaging defensive microbes and pathogens through host populations, we found evidence of defensive microbe–pathogen co‐evolution via fluctuating selection dynamics.

We detected defensive microbe–pathogen interaction specificity as a result of experimental co‐passage. Such specificity is a key assumption underlying co‐evolution by fluctuating selection dynamics (Agrawal & Lively 2002). At passage 10, all replicate pathogen populations were locally adapted in time to the defensive microbe, whilst the defensive microbe was locally maladapted, resulting in the appearance that the pathogen was ‘winning’. This pattern may parallel studies of host–pathogen co‐evolution which have found that pathogen local adaptation is more commonly identified than host local adaptation because pathogens have faster evolutionary responses to reciprocal adaptation than hosts (i.e. due to higher rates of pathogen migration and mutation, or shorter generation times) (Gandon *et al*. 2008; Koskella 2014). Although our experimental design did not allow for migration, we observed that all pathogen populations accumulated more mutations than defensive microbe populations, suggesting a faster pathogen evolutionary rate.

By performing time‐shift experiments, we found that both pathogens and defensive microbes were constantly and reciprocally adapting to each other throughout the experiment. We show that both species peaked in fitness against antagonist populations recently experienced in evolutionary history, a pattern expected from reciprocal evolutionary change. Here, the fitness of a focal species against their recent enemy is expected to be higher than against their current enemy as the current enemy has begun to respond to the adaptive changes of the focal species (Koskella 2014). Critically, our time‐shift data also indicate the dynamics of co‐evolution. In line with our finding of interaction specificity, we show that pathogen and defensive microbe adaptation appeared to fluctuate over time, rather than increase directionally, indicating fluctuating selection dynamics (Agrawal & Lively 2002; Gaba & Ebert 2009). For example, the pathogen and defensive microbe genotypes that were common at passage 4 became rare throughout passages 5–7 and then increased in frequency again by passage 8, pointing to a full cycle of genotype fluctuation.

Consistent with our phenotypic evidence of fluctuating selection dynamics, we found direct confirmation of rapid genotype frequency changes across the evolution experiment. We revealed that mutations that had spread rapidly in the population by passage 5 were almost all rare (or extinct) by passage 10, whilst mutations that were rare (or nonexistent) at passage 5 increased in frequency by passage 10. Importantly, many of these mutations were located in genes putatively linked to *Staphylococcus aureus–Enterococcus faecalis* interactions, including siderophore production by *S. aureus* (Ford *et al*. 2016) and superoxide production by *E. faecalis* (King *et al*. 2016). Kwiatkowski *et al*. (2012) show that such oscillations in defensive microbe and pathogen alleles are likely to occur when the host genetic background is evolutionarily stable, as in our experiment. This outcome suggests that defensive microbes could ‘take over’ from the host and provide rapid co‐evolutionary responses to pathogens (Kwiatkowski *et al*. 2012), which could remove or reduce parasite‐mediated selective pressure on hosts (Hahn & Dheilly 2016; Martinez *et al*. 2016). We speculate that possessing a defensive microbe might allow hosts with relatively limited evolutionary potential (e.g. due to asexual reproduction, low genetic diversity or long generation times) to maintain resistance to co‐evolving pathogens.

An important implication of our findings is that defensive microbes have the potential to be a major source of selection on pathogens, driving fast evolutionary rates and divergent evolutionary trajectories. We observed here that pathogens co‐evolving with defensive microbes had higher rates of evolution, greater genetic turnover and more diverse evolutionary trajectories than those evolving independently in host populations. Similar patterns of molecular evolution in pathogens have been found to occur during host–pathogen co‐evolution (Paterson *et al*. 2010; Schulte *et al*. 2010). Unlike the pathogen, however, we found no difference in the rate of genomic evolution between the co‐passaged and independently passaged defensive microbe populations. It is possible that we saw such asymmetry because *S. aureus* was ahead in the co‐evolutionary arms race and, according to our time‐shift data, was starting a new cycle to which the defensive microbe was not yet adapted to. It is therefore likely that more evolutionary changes had accumulated in co‐evolved *S. aureus* populations relative to independently passaged populations, whilst this was not yet the case for *E. faecalis*. This explanation is consistent with our observations of pathogen local adaptation and the higher total number of mutations in the pathogen populations compared to the defensive microbe populations. Given that co‐evolution started de novo, we would expect that more evolutionary time, and so more cycles of reciprocal adaptation, would eventually result in greater evolutionary change in both species relative to the independently passaged populations.

Together, our data show the potential for defensive microbes and pathogens to engage in co‐evolution driven by fluctuating selection. Our findings suggest that co‐evolution may underlie the interaction specificity observed between pathogens and defensive microbes in nature (Oliver *et al*. 2005; Poulsen *et al*. 2010; Koch & Schmid‐Hempel 2012; Rouchet & Vorburger 2012; Cariveau *et al*. 2014; Cayetano & Vorburger 2015). Given that studies investigating co‐evolution have largely focussed on host–parasite interactions (Agrawal & Lively 2002; Decaestecker *et al*. 2007; Gandon *et al*. 2008; King *et al*. 2009; Morran *et al*. 2011), it is an implied assumption that the host is the strongest source of selection acting upon parasites and pathogens. Although we do not account for additional effects of host immunity on *E. faecalis–S. aureus* interactions in this study, it is nevertheless clear that defensive microbes can be as powerful as hosts in imposing strong and divergent selection on pathogens. Identifying that pathogens are able to undergo co‐evolution with defensive microbes, instead of the host, has important implications for how we understand the factors shaping patterns of pathogen evolution in nature. Moreover, the ability of defensive microbes to co‐evolve with pathogens may have great consequences for their role in disease control. For example, if defensive microbes are able to undergo continual counteradaptation to pathogen resistance, they could be powerful tools to sustainably control infectious diseases of animals and plants (Levin & Bull 2004; Ford & King 2016). In an era of emerging antibiotic resistance, the allure of evolution‐proof disease control is at an all‐time high (Allen *et al*. 2014; Vale *et al*. 2016).

It is being increasingly revealed that defensive microbes are common within host populations and across numerous host species. As a result, we are only just beginning to realize their immense value to hosts. In this study, we further our understanding of defensive microbes by illustrating their role as an evolutionarily dynamic arm of host immunity and their role in driving pathogen divergence.

## Data accessibility

Raw read data for the bacterial genomic sequences were deposited in the European Nucleotide Archive under project accession no. PRJEB13385. The CSV files containing data for each figure are available in the Dryad repository (http://dx.doi.org/10.5061/dryad.0b707) (Ford *et al*. 2016).

S.A.F. and K.C.K. designed the experiments. S.A.F. conducted the experiments and analysed the data. S.A.F., D.W. and S.P. conducted the bioinformatics. S.A.F. and K.C.K. wrote the manuscript.

## Supporting information


**Fig. S1** (a) Fitness of *Enterococcus faecalis* (CFU/host) and (b) *Staphylococcus aureus* (CFU/host) under sympatric and allopatric‐in‐time conditions (Single evolution treatment).
**Table S1** Statistical results listed by figure.

**Appendix S1** Mutations found present in replicate *E. faecalis* genomes across all treatments.

**Appendix S2** Mutations found present in replicate *S. aureus* genomes across all treatments.
