# Supplementary material for: Co‐evolutionary dynamics between a defensive microbe and a pathogen driven by fluctuating selection
Source: Mol Ecol. 2016 Dec 5;26(7):1778–89. doi: 10.1111/mec.13906 (PMC6849518; doi:10.1111/mec.13906)
Supplement: Supplementary file 1 — Fig. S1 (a) Fitness of Enterococcus faecalis (CFU/host) and (b) Staphylococcus aureus (CFU/host) under sympatric and allopatric‐in‐time conditions (Single evolution treatment). Table S1 Statistical results listed by figure. [file MEC-26-1778-s001.docx]

**Supporting figure 1**

a) Fitness of *E. faecalis* (CFU/host) and (b) *S. aureus* (CFU/host) under sympatric and allopatric-in-time conditions (Single evolution treatment).

| Figure  **Supporting Table 1.** Statistical results listed by figure. | Statistic |
| --- | --- |
| 1b | **ANOVA:** F=7.98, df=2, P=0.0063  **Tukey Contrasts:**  Coevolution-Ancestor: P=0.008  Single evolution-Ancestor: P=0.86  Single evolution-Coevolution: P=0.02 |
| 1c | **ANOVA**: F=4.86, df=2, P=0.028  **Tukey Contrasts:**  Coevolution-Ancestor: P=0.071  Single evolution-Ancestor: P=0.91  Single evolution-Coevolution: P=0.034 |
| 2b | **Mixed-effect model** with replicate population as random effect and sympatric/allopatric treatment as fixed effect.  Chisq=5.82, df=1, P=0.016 |
| 2c | **Mixed-effect model** with replicate population as random effect and sympatric/allopatric treatment as fixed effect.  Chisq=5.79, df=1, P=0.016 |
| 3c | **Mixed-effect model** with replicate population as random effect and *E. faecalis* passage as fixed effect.  Chisq=29.1, df=6, P=5.819e-05  **Tukey Contrasts:**  5-8: P<0.001  4-8: P=0.99  9-8: P=0.99  7-8: P=0.31  6-8: P=0.001  10-8: P=0.99  4-5: P<0.001  9-5: P<0.001  7-5: P=0.28  6-5: P=29  10-5: P<0.001  9-4: P=1  7-4: P=0.079  6-4: P=0.005  10-4: P=0.99  7-9: P=0.14  6-9: P=0.017  10-9: P=0.99  6-7: P=0.99  10-7: P=0.025  10-6: P=0.0028 |
| 3d | **Mixed-effects model** with replicate population as random effect and *S. aureus* passage as fixed effect.  Chisq=17.18, df=6, P=0.0086  **Tukey Contrasts:**  5-8: P=0.011  4-8: P=0.99  9-8: P=0.017  7-8: P=0.84  6-8: P=0.14  10-8: P=0.70  4-5: P=0.034  9-5: P=1  7-5: P=0.36  6-5: P=0.98  10-5: P=0.52  9-4: P=0.048  7-4: P=0.95  6-4: P=0.28  10-4: P=0.88  7-9: P=0.43  6-9: P=0.99  10-9: P=0.60  6-7: P=0.88  10-7: P=1  10-6: P=0.95 |
| 5a | Pairwise distances of passage 10 from ancestor, comparing Coevolution and Single evolution treatments:  **Two Sample t-test** t = -2.95, df = 8, P= 0.018  **fdr-corrected P=** 0.018  Within-treatment pairwise distances of passage 10, comparing Coevolution and Single evolution treatment:  **Welch two Sample t-test** t = -6.97, df = 12.9, P=1.036e-05  **fdr-corrected P=** 3.108e-05  Pairwise distances of passage 5 to passage 10 replicate populations, comparing Coevolution and Single evolution treatments:  **Wilcoxon rank sum test** W = 25 , P= 0.0079  **fdr-corrected P=** 0.012 |
| 5b | Pairwise distances of passage 10 from ancestor, comparing Coevolution and Single evolution treatments:  **Two Sample t-test** t = 1.1, df = 8, P= 0.302  **fdr-corrected P=** 0.302  Within-treatment pairwise distances of passage 10, comparing Coevolution and Single evolution treatments:  **Two Sample t-test** t = 2.36, df = 18, P= 0.03  **fdr-corrected P=** 0.09  Pairwise distances of passage 5 to passage 10 replicate populations, comparing Coevolution and Single evolution treatments:  **Two Sample t-test** t = 1.55, df =8, P= 0.16  **fdr-corrected P=** 0.24 |
| Supporting figure 1a | **Mixed-effect model** with replicate population as random effect and sympatric/allopatric treatment as fixed effect.  Chisq=1.8, df=1, P=0.18 |
| Supporting figure 1b | **Mixed-effect model** with replicate population as random effect and sympatric/allopatric treatment as fixed effect.  Chisq=0.8, df=1, P=0.36 |
